# Effects of different three-dimensional electrodes on epiretinal electrical stimulation by modeling analysis

**DOI:** 10.1186/s12984-015-0065-x

**Published:** 2015-08-28

**Authors:** Xun Cao, Xiaohong Sui, Qing Lyu, Liming Li, Xinyu Chai

**Affiliations:** School of Biomedical Engineering, Shanghai Jiao Tong University, No.800 Dongchuan Road, Shanghai, People’s Republic of China

## Abstract

**Background:**

Epiretinal prostheses have been greatly successful in helping restore the vision of patients blinded by retinal degenerative diseases. The design of stimulating electrodes plays a crucial role in the performance of epiretinal prostheses. The objective of this study was to investigate, through computational modeling analysis, the effects on the excitation of retinal ganglion cells (RGCs) when different three-dimensional (3-D) electrodes were placed in the epiretinal space.

**Methods:**

3-D finite element models of retinal electrical stimulation were created in COMSOL using a platinum microelectrode, a vitreous body, multi-layered retinal tissue, and retinal pigment epithelium (RPE). Disk and non-planar electrodes with different 3-D structures were used in the epiretinal electrical stimulation. In addition, a multi-RGC model including ionic mechanisms was constructed in NEURON to study the excitability of RGCs in response to epiretinal electrical stimulation by different types of electrodes. Threshold current, threshold charge density, and the activated RGC area were the three key factors used to evaluate the stimulating electrode’s performance.

**Results:**

As the electrode-retina distance increased, both threshold current and threshold charge density showed an approximately linear relationship. Increasing the disk electrode’s diameter resulted in an increase in threshold current and a decrease in threshold charge density. Non-planar electrodes evoked different activation responses in RGCs than the disk electrode. Concave electrodes produced superior stimulation localization and electrode safety while convex electrodes performed relatively poorly.

**Conclusions:**

Investigation of epiretinal electrical stimulation using different 3-D electrodes would further the optimization of electrode design and help improve the performance of epiretinal prostheses. The combination of finite element analysis in COMSOL and NEURON software provides an efficient way to evaluate the influences of various 3-D electrodes on epiretinal electrical stimulation. Non-planar electrodes had larger threshold currents than disk electrodes. Of the five types of electrodes, concave hemispherical electrodes may be the ideal option, considering their superior stimulation localization and electrode safety.

## Background

Vision is the most important neural system for human beings. However, some retinal degenerative diseases such as retinitis pigmentosa (RP) and age-related macular degeneration (AMD) can lead to profound blindness, which is incurable by current medical therapy [1]. These two diseases result in a massive and irreversible loss of photoreceptors while a large number of inner retinal neurons survive [2].Visual prosthesis provides a promising approach for restoring functional vision through electrical stimulation of the surviving neural tissues in the visual pathway (visual cortex [3], lateral geniculate nucleus [4], optic nerve [5], or retina [6]).

The Second Sight Argus™ II epiretinal prosthesis has been authorized by the European CE and USA FDA [7, 8]. Subjects implanted with Argus™ II could perform tasks including door finding, tracking line orientation, and spatial-motor detection [9, 10]. The stimulating electrode array in the epiretinal prosthesis is placed on the inner surface of the retina in the macular region, close to retinal ganglion cells (RGCs) [6, 11]. It elicits punctate phosphenes by electrically stimulating the surviving inner retinal neurons (bipolar cells and/or RGCs) to produce artificial vision. According to clinical trials, subjects implanted with an epiretinal prosthesis described the phosphenes as round or oval spots [12]. However, previous experiments also included reports of irregular punctate perceptions, including donut shapes, lines, and clusters of dots [6, 13–15]. A mapping distortion of phosphenes in response to regular electrode array stimulation was also reported [16–18]. This may due to the complicated structure of the human retina. There are 5 to 7 layers of RGCs distributing in the macular region non-uniformly and the optic nerve fiber (i.e. RGC axon) layer is located above the RGC somata, closer to the inner surface of the retina [19]. When the electrode array stimulates the retina, the passing axons of RGCs may be activated, which would produce irregular phosphenes [20]. To produce regular phosphenes, improving spatial stimulation localization is necessary. Thus, it is important to investigate the responses of RGCs to electrical stimulation.

Several computational RGC models have been reported in previous studies. Greenberg et al. [21] used three kinds of neuronal membrane models of RGCs: a linear passive model, a Hodgkin-Huxley (H-H) model with passive dendrites, and a model composed of five nonlinear ion channels (Fohlmeister-Coleman-Miller model). They reported that the site with the lowest RGC excitation threshold for electrical stimulation was over the soma. In consideration of an RGC’s structural features, Schiefer et al. [20] revised Greenberg’s RGC model and added an approximately 90° bend in the RGC axon near its point of attachment to the soma. It turned out that the threshold to excite one RGC was lowest when the electrode was positioned near the bend rather than over other parts of the axon. Fried et al. [22] found a dense band of voltage-gated sodium channel located in the axon not far from the soma in rabbit ganglion cells. The lowest threshold to excite the RGC was found within this band, which indicated that this sodium-channel band was the site most sensitive to external electrical stimulation. Jeng et al. [23] built a computational neuron model to study the influence of the sodium channel band on electrical stimulation. Mueller et al. [24] developed a population neuron model to analyze multi-electrode stimulation of RGCs.

In the electrical stimulation modeling work mentioned above, all the stimulating electrodes were either point source electrodes or disk electrodes. Disk electrode arrays showed limited stimulation localization in RGCs [25]. As a result, different non-planar stimulating electrode models have been proposed in previous studies. Rattay et al. [26] constructed long electrodes that were positioned parallel to the axons of RGCs. Their results proved this type of electrode was more effective at avoiding co-stimulation of the somata and passing axons. Djilas et al. [27] proposed a well-shaped three-dimensional (3-D) electrode, which optimized the localization of electrical stimulation. In addition, there are many other kinds of non-planar electrode designs that have been proposed. Thus, it is necessary to determine which type of electrode would be the ideal candidate for epiretinal stimulation.

The objective of this study was to determine how different 3-D electrodes positioned in the epiretinal space would affect RGC excitation through the use of computational analysis. The aim is to provide a useful understanding of different stimulating electrode parameters and to give some guidance on future electrode designs. For disk electrodes, we studied the impact of electrode-retina distance (ERD) and electrode size on RGC activation. Furthermore, we investigated the influence of the geometrical shape of electrodes, including convex hemispherical, convex conical, concave hemispherical, and concave conical electrodes. A multi-layered retinal stimulation model was created using the finite-element-method (FEM) to compute the external electric field, and a multi-RGC model with ionic mechanisms was built to detect whether RGCs were activated by the electrical stimulation.

## Methods

### Retinal stimulation model

**Fig. 1:**
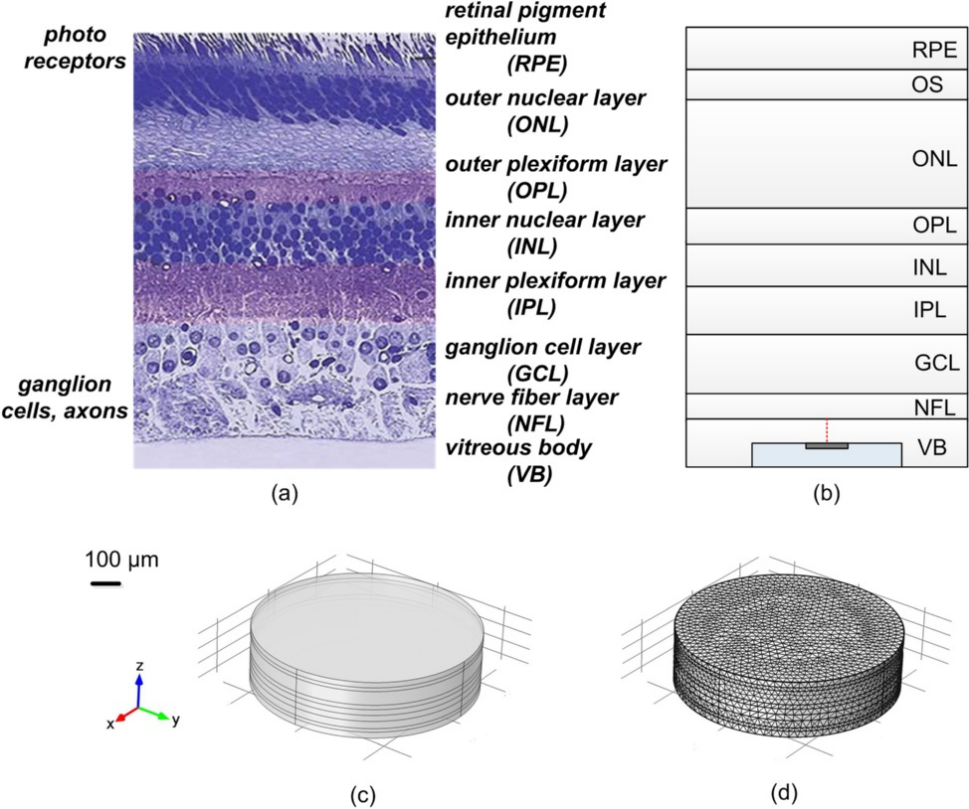
The structure of the retinal model. **a** Histological structure of human retina (image adapted from Ref. [42] with permission) (**b**) Schematic diagram of the retinal computational model in COMSOL (**c**) Semi-transparent view of the 3-D FEM retinal model (**d**) View of the 3-D FEM retinal model in tetrahedron mesh

The retinal stimulation model was comprised of a multi-layered retinal model and a stimulating electrode model. Figure 1 shows the schematic of the retinal stimulation model, consisting of a microelectrode, vitreous body, and the retina. The entire model was cylindrical (Fig. 1(b)), with a diameter of 2.8 mm and a height of 372–432 μm, varying with the ERD. These computational models were constructed using the AC/DC module in COMSOL Multiphysics 4.3 (COMSOL AB, Sweden).

#### Multi-layered retinal model

The multi-layered retinal model included a retina portion and a vitreous body portion. The retina portion consisted of the nerve fiber layer (NFL), ganglion cell layer (GCL), inner plexiform layer (IPL), inner nuclear layer (INL), outer plexiform layer (OPL), outer nuclear layer (ONL), outer segment (OS) of photoreceptors, and retinal pigment epithelium (RPE) (Fig. 1(b)). The vitreous body was located between the stimulating electrode and retinal surface, and its thickness changed with the ERD marked by the dotted red line (Fig. 1(b)). The thickness and conductivity parameters of each layer were determined by the anatomical structure of the human retina and previous modeling studies (Fig. 1(a)) [28, 29]. All the parameters are listed in Table 1.

**Table 1:** Parameters of the multi-layered retinal model

Component		Conductivity (S/m)	Thickness (μm)
Microelectrode	Metal: Pt	8.9 × 10^6^	1
	Substrate: PI	1.0 × 10^−17^	20
Interface	Vitreous body	1.2821	20-80
Retinal layers	NFL	0.0126[28]	24[29]
	GCL	0.0126[28]	49[29]
	IPL	0.0571[28]	40[29]
	INL	0.0147[28]	38[29]
	OPL	0.0571[28]	30[29]
	ONL	0.0147[28]	91[29]
	OS	1.0309[28]	25[29]
	RPE	0.0010[28]	35[29]

[28] Zhou D D and Greenbaum E, 2009

[29] Van Dijk H W, Kok P H,et al., 2009

#### Stimulating electrode model

**Fig. 2:**
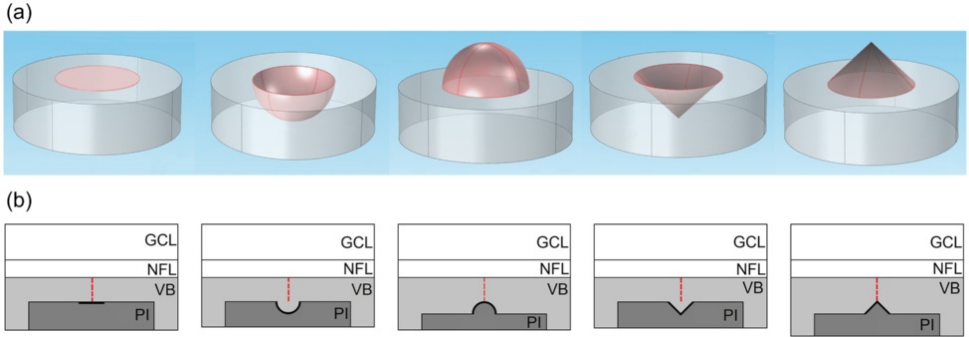
Stimulating electrode models. From left to right: disk electrode, concave hemispherical electrode, convex hemispherical electrode, concave conical electrode, convex conical electrode. **a** Five different spatial geometrical structures of stimulating electrodes (**b**) Schematic diagrams of the spatial placement of electrodes. Red dotted lines represent the ERD

A platinum (Pt) stimulating electrode model based on an insulating polyimide (PI) substrate was positioned below the retinal tissue. Due to the curved structure of the human retina, it is difficult to achieve flush contact between the electrode and retina [8]. Here, the ERD was defined as the shortest distance between the stimulating electrode and the inner surface of the retinal tissue (red dotted lines in Fig. 2(b)) and varied from 20 to 80 μm in increments of 20 μm. Due to the fact that smaller electrodes may produce a better spatial resolution, disk electrodes with diameters (Φ) ranging from 50 to 200 μm in 50 μm increments were studied. In order to study the effects of an electrode’s geometrical shape on RGC activation, four non-planar electrodes were modeled: convex hemispherical, convex conical, concave hemispherical, and concave conical electrodes (Fig. 2(a)). All the non-planar electrodes had a 100 μm diameter (Φ = 100 μm) disk projection to the retinal plane. The conical electrodes were right-angle cones with a height of 50 μm. The return electrode for electrical stimulation was assumed to be at a distance of infinity, simulating monopolar electrical stimulation. The stimulating current was a cathodic monophasic stimulating current pulse with a fixed duration of 0.4 ms, similar to previous studies [24].

### Retinal ganglion cell excitation model

To investigate the response of RGC’s to different electrical stimulation, both single-RGC and multi-RGC models were developed using NEURON [30].

#### Single RGC model

**Fig. 3:**
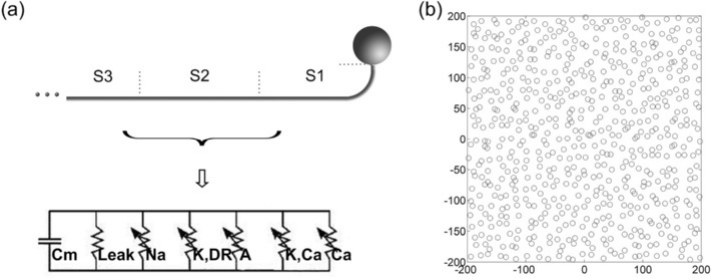
Retinal ganglion cell excitation models. **a** Schematic diagram of a single RGC model. **b** Top view of the 400 μm × 400 μm × 50 μm space with 625 randomly located RGCs, and each circle represents the soma of the RGC

The parameters of the ion channels in our RGC model were derived from tiger salamander data [31]. Our model included five active membrane currents: sodium current I_Na_, delayed-rectifier potassium current I_K,DR_, A-type rapidly inactivating potassium current I_A_, calcium-activated potassium current I_K,Ca_, and T-type calcium current I_Ca_. A leakage current was included as well [20, 23]. To better simulate the morphology and function of RGCs, a 90° bend [20, 24] and a segment with a high-density sodium-channel band about 40 μm from the soma [22, 23] were added (Fig. 3(a)). The axon hillock segment (S1 in Fig. 3(a)) was the portion emerging immediately from the soma; it included a 0.5 μm vertical extension, a 90° bend with a radius of 5 μm, and a 40 μm horizontal extension. The sodium channel segment (S2 in Fig. 3(a)), with a 40 μm long high-density sodium-channel band, was connected to S1 and the distal axon segment (S3 in Fig. 3(a)). The distal axon segment extended 1000 μm from the soma horizontally. The ion channel conductances of each segment were adopted from Sheasby and Fohlmeister [31]. All the active ion channels were modeled to be voltage-gated except I_K,Ca_, which depended on the level of intracellular calcium. The membrane potential E follows Kirchoff’s law:

$$ \begin{array}{l}{C}_mdE/dt=-{\widehat{G}}_{Na}{m}^3h\left(E-{E}_{Na}\right)-{\widehat{G}}_{Ca}{c}^3\left(E-{E}_{Ca}\right)\\ {}\kern1em -{\widehat{G}}_{K,DR}{n}^4\left(E-{E}_K\right)-{\widehat{G}}_A{a}^3{h}_A\left(E-{E}_K\right)\\ {}\kern1em -{\widehat{G}}_{K,Ca}\left(E-{E}_K\right)-{\widehat{G}}_1\left(E-{E}_1\right)\end{array} $$

where *E* stands for the membrane potential, *E*_Na_, *E*_K_, *E*_Ca_, and *E*_l_ are the equilibrium potentials for the corresponding ion channels, and *Ĝ*_*Na*_, *Ĝ*_*Ca*_, *Ĝ*_*K*,*DR*_, *Ĝ*_*A*_, *Ĝ*_*K*,*Ca*_, and *Ĝ*_1_ represent the maximum conductance value of each ion channel. All these parameters can be seen in Table 2. The dimensionless gating variables *m, h, c, n, a,* and *h*_*A*_, follow the equation below [23]:

**Table 2:** Parameters of Morphology and conductance for the single RGC model

	Axon			Soma
	S1	S2	S3	
Diameter (μm)	3	3	0.9	10
Length (μm)	48	40	1000	
*Ĝ*_*Na*_ (*E*_*Na*_ = +35 mV)	70	350	100	80
*Ĝ*_*K*,*DR*_ (*E*_*K*_ = −75 mV)	18	45	18	18
*Ĝ*_*A*_ (*E*_*K*_ = −75 mV)		54	54	54
*Ĝ*_*Ca*_ (*E*_*Ca*_ = 132 mV)		1.5		1.5
*Ĝ*_*K*,*Ca*_	0.065	0.065	0.065	0.065
*Ĝ*_1_	0.005	0.005	0.005	0.005

$$ dx/dt=-\left({\alpha}_x+{\beta}_x\right)\cdot x+{\alpha}_x $$

where *α* and *β* are rate constants that have dimensions of [time]^−1^ and are determined by membrane potential (Table 3). The membrane capacitance, *C*_*m*_, was fixed at 1 μF cm^−2^.

**Table 3:** Dynamic equations for ionic channels

Ion	Channel	State variable	*α*	*β*
Na	Fast sodium	*m*	$\frac{-0.6*\left(\mathrm{V}+30\right)}{e^{-0.1*\left(\mathrm{V}+30\right)}-1}$	$\frac{20}{e^{\frac{\mathrm{V}+55}{18}}}$
		*h*	$\frac{0.4}{e^{\frac{\mathrm{V}+50}{20}}}$	$\frac{6}{1+{e}^{-0.1*\left(\mathrm{V}+20\right)}}$
K	Delayed	*n*	$\frac{-0.02*\left(\mathrm{V}+40\right)}{e^{-0.1*\left(\mathrm{V}+40\right)}-1}$	$\frac{0.4}{e^{\frac{\mathrm{V}+50}{80}}}$
	A-type	*a*	$\frac{-0.006*\left(\mathrm{V}+90\right)}{e^{-0.1*\left(\mathrm{V}+40\right)}-1}$	$\frac{0.1}{e^{\frac{\mathrm{V}+30}{10}}}$
		*h*_*A*_	$\frac{0.04}{e^{\frac{\mathrm{V}+70}{20}}}$	$\frac{0.6}{e^{-0.1*\left(\mathrm{V}+90\right)}+1}$
	Calcium		${I}_{K,Ca}={\widehat{G}}_{K,Ca}\left(\frac{{\left[Ca\right]}_i/0.001}{1+{\left[Ca\right]}_i/0.001}\right)\left(\mathrm{V}-{e}_k\right)$	
Ca	Ca	*c*	$\frac{-0.3*\left(\mathrm{V}+13\right)}{e^{-0.1*\left(\mathrm{V}+13\right)}-1}$	$\frac{10}{e^{\frac{\mathrm{V}+38}{18}}}$

### Multi-RGC model

The single RGC model was replicated 625 times to build the multi-RGC model. In the multi-RGC model, RGCs were randomly distributed in a space of 400 μm × 400 μm × 50 μm (Fig. 3(b)). The rand function in MATLAB was used to create 625 points representing the centers of the RGC somata in this space. This RGC density was selected according to a histological report on the human macula [32]. Since the multi-RGC model simulated the macular RGCs of the right human eye, all the RGC axons extended left toward the nasal side when they emerged from the somata. The multi-RGC model was used to study the influence of different electrical stimulation on RGCs. The responses of the ganglion cells were evaluated by three factors: the threshold current (TC) – the minimum stimulation current that could activate a single RGC; threshold charge density (TCD) - the minimum charge density that could activate a single RGC; and the activated RGC area – the region of the activated RGCs under a certain electrical stimulation condition.

### Integration of the retinal stimulation model and the retinal ganglion cell excitation model

The retinal stimulation model implemented in COMSOL was integrated with the RGC excitation model developed in NEURON. Stimulating currents with different intensities were assigned to the 3-D electrode models in COMSOL, and electric fields were generated inside the multi-layered retinal model. Then the electric voltages computed in COMSOL were exported to NUERON as the extracellular voltages of our multi-RGC model.

To increase the simulation accuracy of this study, an extremely fine mesh strategy was used in COMSOL. The whole model was meshed with a maximum element size of 13.2 μm, but the mesh segmentation was refined at the NFL and GCL using the “split longest side” method, ensuring that each element size was less than 1 μm. Compared with the multi-RGC model in NEURON, where the size of each segment was 1 μm, the meshing size in COMSOL in the stimulated area of the retina was smaller and more precise. Thus, when we imported the data from COMSOL into NEURON, no accuracy was lost. To aid understanding of the mesh strategy used in COMSOL, a specific example is presented as follows: for a Φ = 50 μm disk electrode with an ERD = 20 μm model, there were 31572 elements meshed in the platinum material electrode. The NFL & GCL layers consisted of 841756 elements, and there were 3304362 elements in total.

## Results

### Spatial distribution of the electric field in the GCL

**Fig. 4:**
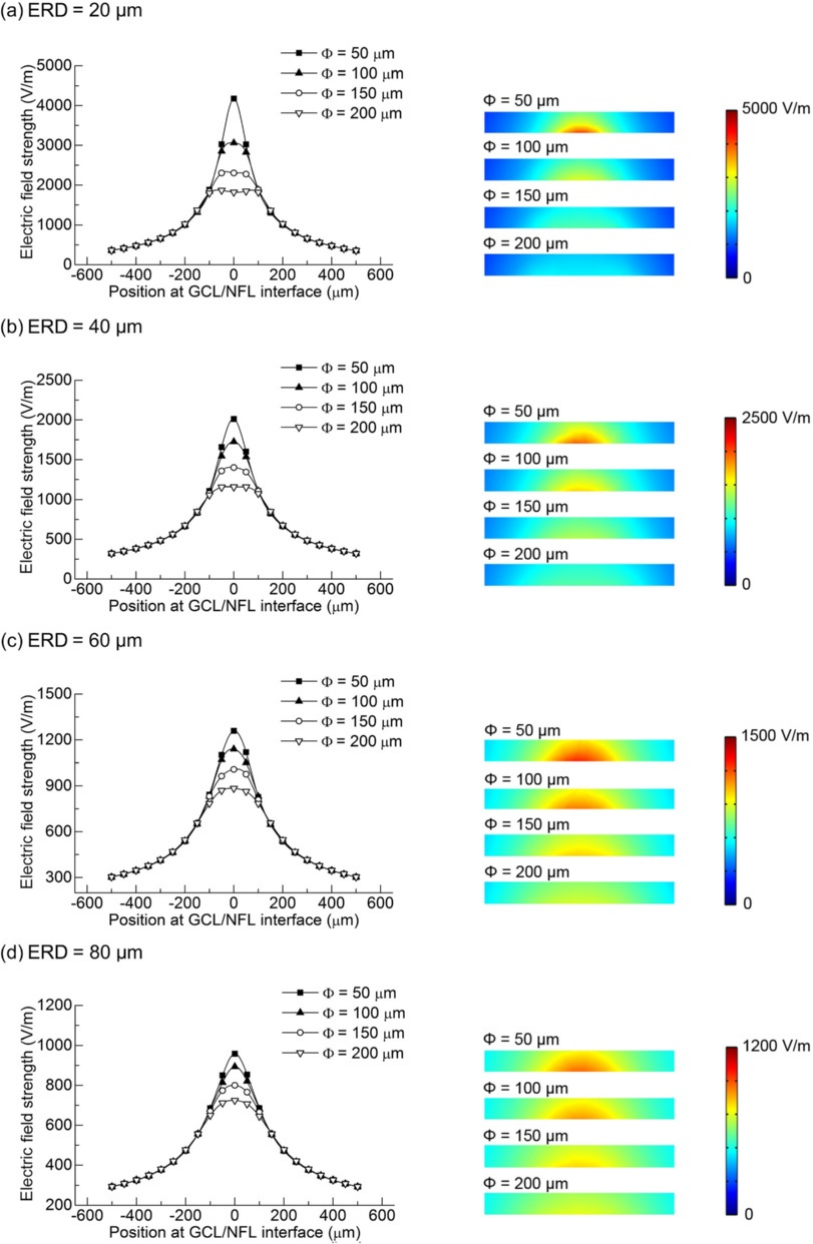
The electric field distribution in GCL of different disk electrodes with 20 μA cathodic stimulus. Left: the electric field strength at the surface of GCL; Right: the general electric field distribution in GCL shown in COMSOL. **a** ERD = 20 μm. **b** ERD = 40 μm. **c** ERD = 60 μm. **d** ERD = 80 μm

**Fig. 5:**
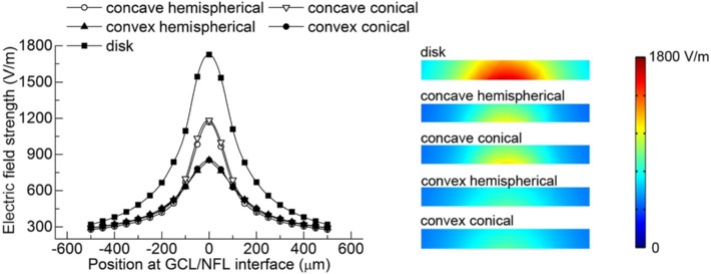
The electric field distribution in GCL of different electrode shapes with 20 μA cathodic stimuli. Left: the electric field strength at the surface of GCL; Right: the general electric field distribution in GCL shown in COMSOL. All the stimulating electrodes had the same projection of Φ = 100 μm disk to the retinal plane and an ERD of 40 μm

The electrode size, geometrical shape, and ERD showed an obvious impact on the spatial distribution of the electric field in the retina. Cross-sectional views of the electric field distributions in the GCL are shown in Figs. 4 and 5.

#### Same-amplitude current stimulation for disk electrodes

A 20 μA cathodic stimulus was applied to disk electrodes with different diameters (Φ = 50, 100, 150, 200 μm) at different electrode-retina distances (ERD = 20, 40, 60, 80 μm). The electrical field distribution results are shown in Fig. 4.

The maximum electric field strength appeared in the central area or the perimeter area above the disk electrodes. For smaller electrodes, the maximum field strength was in the central area of the GCL axially above the electrode surface (Fig. 4(a) Φ = 50 μm). However, as the diameter increased, the maximum field strength decreased dramatically, and the electric field distribution became more uniform. For example, the maximum field strength for a 200 μm diameter electrode was only half the value of the maximum field strength for a 50 μm diameter electrode. In addition, for larger electrodes, two peaks in electric field strength appeared above the perimeter area of electrodes (Fig. 4(a) Φ = 200 μm), which was caused by the edge effect of the micro-electrode. For a given electrode diameter, the maximum electric field strength decreased dramatically as the ERD increased. For a Φ = 50 μm disk electrode, the value decreased from 4176 V/m to 958 V/m as the ERD increased from 20 μm to 80 μm (Fig. 4(a)-(d)). The difference in electric field strength for small and large electrodes became smaller and smaller with the increase of ERD (Fig. 4(d)). Also, the electrode edge effect weakened as the ERD increased.

#### Same-amplitude current stimulation for non-planar electrodes

A 20 μA cathodic stimulus was applied to four non-planar electrodes as well as a disk electrode with Φ = 100 μm and ERD = 40 μm (Fig. 5).

Compared with the disk electrode, the electric field strengths of the four non-planar electrodes were lower under the same electrical stimulation conditions. Because the disk electrode had the smallest surface area, its stimulating charge density was the largest, and thus it produced a stronger electric field than the other electrodes. The peaks in electric field strength of the non-planar electrodes were narrower and lower than those of the disk electrode. The conical and hemispherical electrodes with matching concavity or convexity had similar electric field strength distributions. However, quantitatively speaking, for both the concave and convex electrodes, the maximum electric field strengths of the conical electrodes were 2 % higher than those of the hemispherical electrodes. In addition, the concave and convex electrodes had similar electric field strengths at the outer electrode perimeter area, but in the central area the electric field strengths of the concave electrodes were 1.5 times those of the convex electrodes.

### Effects of electrode position on threshold profile for single RGC model

In previous electrophysiological experiments, an RGC showed the lowest activation threshold when the stimulation electrode was placed over the high-density sodium-channel band [22]. To validate our RGC model against these results, we quantified the threshold for exciting the single RGC model by using a Φ = 100 μm disk electrode and varying the electrode location along the axon in NEURON.

**Fig. 6:**
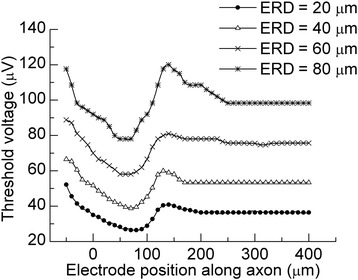
Profile of the threshold to excite a single RGC model at different ERDs. The disk electrode had a 100 μm diameter

Threshold voltages are shown in Fig. 6 as a function of stimulating electrode position along the axon. In accordance with previous studies [22, 23], the lowest thresholds were found not directly above the soma (at zero), but rather when the stimulating electrode was passing over the high-density sodium-channel band (between 40 μm and 80 μm). Thresholds increased with the increase of ERD, but the threshold profile at each ERD remained similar.

### Responses of the multi-RGC model to stimulation with disk electrodes

**Fig. 7:**
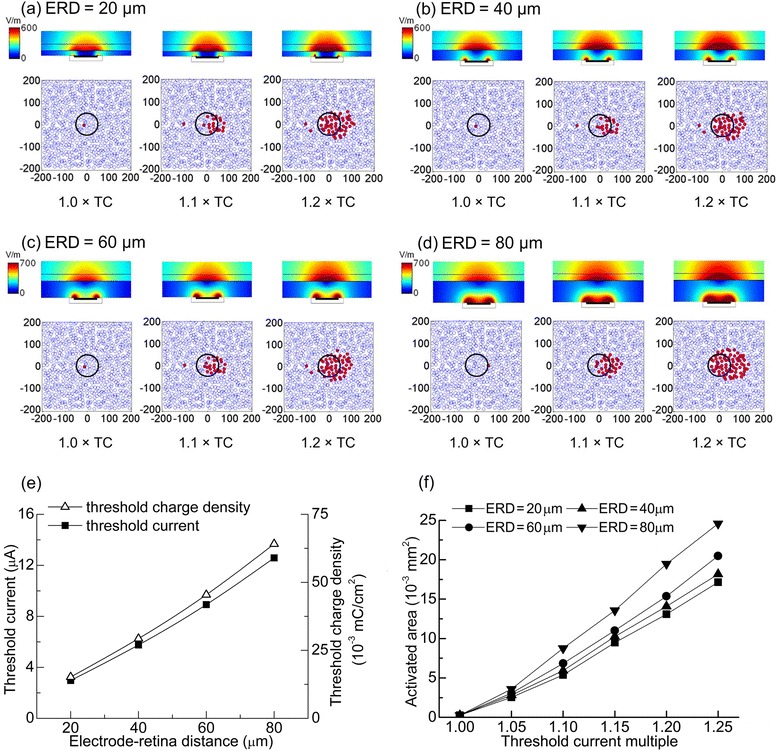
Responses to stimulation by 100 μm diameter disk electrodes at different ERDs. **a-d** Qualitative results under the stimulation of 1.0 ×, 1.1 × and 1.2 × TC. Top: the electric field distribution in the retina; Bottom: the activating results of RGCs. Red points meant activated RGCs, while blue ones meant non-activated RGCs. **a** ERD = 20 μm. **b** ERD = 40 μm. **c** ERD = 60 μm. **d** ERD = 80 μm. **e** Correlations among TC, TCD, and the ERD (**f**) Correlation between activated RGC area and stimulating current at different ERDs

**Fig. 8:**
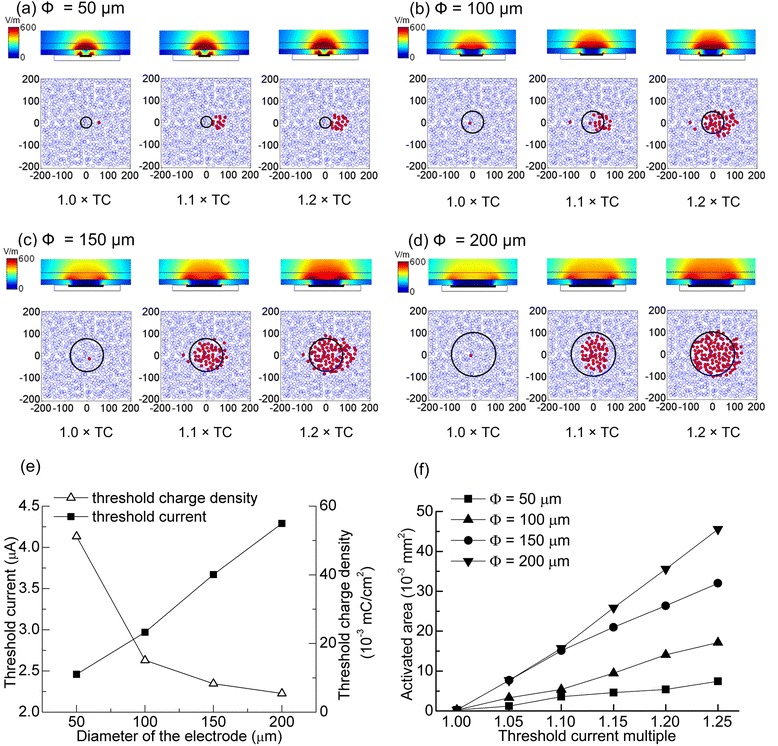
Responses to stimulation with different diameter disk electrodes at ERD = 40 μm. **a-d** Qualitative results under the stimulation of 1.0 ×, 1.1 × and 1.2 × TC. Top: the electric field distribution in the retina; Bottom: the activating results of RGCs. Red points meant activated RGCs, while blue ones meant non-activated RGCs. **a** Φ = 50 μm. **b** Φ = 100 μm. **c** Φ = 150 μm. **d** Φ = 200 μm. **e** Correlations among the TC, TCD, and the electrode size. **f** Correlation between activated RGC area and stimulating current with different electrode sizes

We used the multi-RGC model to analyze the RGC responses to electrical stimulation by disk electrodes. The top views of the responses of RGCs under different epiretinal stimulation are shown in Figs. 7 and 8, in which the somata of the non-activated RGCs are outlined in blue and those of the activated RGCs are highlighted in red. The size and position of the stimulating electrodes are marked with a black circle. In this study, we used the activated RGC area – estimation method as proposed in a previous study [24] – to estimate the effects of electrode parameter variations on electrically evoked RGC responses. To study the activated area of the multi-RGC model under different stimulating currents, values of 1.0, 1.05, 1.1, 1.15, 1.2, and 1.25 times TC were selected. Fig. 7(a)-(d) (top) and Fig. 8(a)-(d) (top) show the electric field distributions in the vitreous body, nerve fiber layer, and ganglion cell layer under the stimulation of 1.0 × TC, 1.1 × TC, and 1.2 × TC; Fig. 7(a)-(d) (bottom) and Fig. 8(a)-(d) (bottom) show the activated RGC areas under those stimulation currents. The centers of the activated RGC areas were not located directly above the stimulating electrodes; instead, there was a small offset in the direction of the axons. Quantitative analyzes of TC, TCD, and activated RGC area are shown in Fig. 7(e), (f) and Fig. 8(e), (f).

#### Effects of ERD on threshold and activated area in GCL

To study the effects of ERD on the threshold and activated RGC area, the electrode size was fixed at Φ = 100 μm. As ERD increased from 20 μm to 80 μm, both the TC and the TCD showed an approximately linear relationship with ERD (Fig. 7 (e)). A linear fitting method was used to fit the TC data to a slope of 1 μA/6.25 μm, which was similar to the threshold values obtained in previous experiments conducted by Sekirnjak et al. [33]. The activated RGC area increased linearly with the increase of stimulating current (Fig. 7(f)). Under the stimulation of 1.25 × TC, the activated RGC area was 0.017 mm^2^ at ERD = 20 μm and increased to 0.025 mm^2^ when ERD = 80 μm.

#### Effects of disk electrode size on threshold and activated area in GCL

To analyze the influence of disk electrode size on RGC activation, ERD was fixed at 40 μm. With Φ increasing from 50 to 200 μm, the TC varied linearly from 2.46 to 4.29 μA. However, the TCD decreased from 0.051 to 0.005 mC/cm^2^ (Fig. 8(e)). The activated RGC area increased approximately linearly as the stimulating current increased (Fig. 8(f)). Under stimulation with the same TC multiple (1.25 × TC), the activated RGC area was 0.007 mm^2^ at Φ = 50 μm and increased to 0.046 mm^2^ when Φ = 200 μm.

### Responses of multi-RGC model to stimulation with non-planar electrodes

**Fig. 9:**
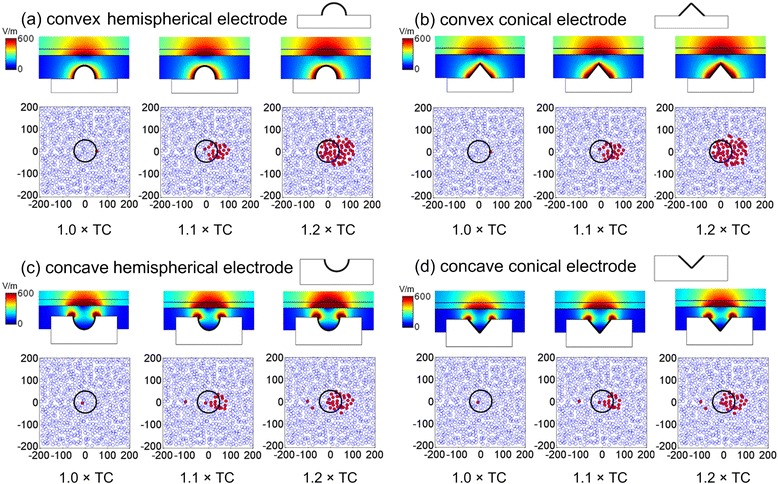
Electric field distributions and activation results for RGCs under stimulation with non-planar electrodes. Top: the electric field distribution in the retina; Bottom: the activating results of RGCs. Red points indicate activated RGCs, while blue ones indicate non-activated RGCs. **a** Convex hemispherical electrode. **b** Convex conical electrode. **c** Concave hemispherical electrode. **d** Concave conical electrode

Four different non-planar electrodes with Φ = 100 μm were used to investigate the responses of the multi-RGC model. The analytical approach for the non-planar electrodes was the same as that for the disk electrodes. The electric field distributions of convex electrodes were more divergent than those of concave electrodes, and the electric field distributions of hemispherical electrodes were quite similar to those of conical electrodes (Fig. 9).

**Fig. 10:**
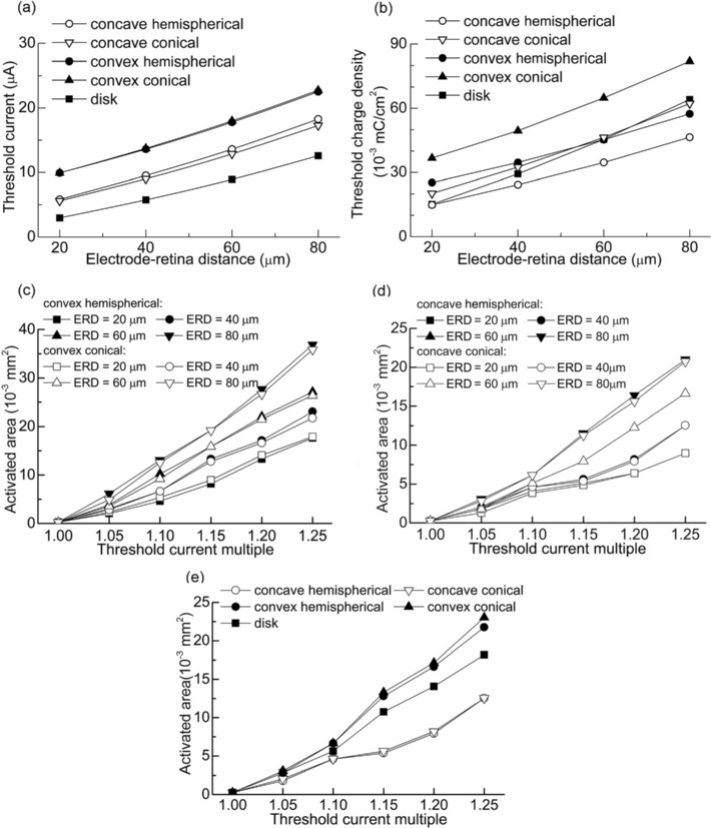
Responses to stimulation of different electrode shapes. **a** Comparisons of TC and the electrode geometrical shape at different ERDs. **b** Comparisons of TCD and the electrode geometrical shape at different ERDs. **c** Correlation between the active RGC areas and stimulating current for convex electrodes. **d** Correlation between the activated RGC area and stimulating current for concave electrodes. **e** Active RGC areas as a function of stimulating current for different electrode shapes at ERD = 40 μm

Quantitative comparisons of TC and TCD for the four non-planar electrodes and the Φ = 100 μm disk electrode are shown in Fig. 10(a), (b). TC and TCD showed a nearly linear relationship with ERD for all electrodes. The disk electrode had the lowest TC, and the convex-shaped electrodes had a higher TC than the concave-shaped electrodes. However, the relative comparisons of TCDs for all electrodes depended on ERDs. With a smaller ERD, the disk and concave hemispherical electrodes had the lowest TCDs. But when ERD increased to 80 μm, the TCDs of the concave hemispherical, concave conical, and convex hemispherical electrodes were all lower than that of the disk electrode. There was an approximately linear correlation between activated RGC area and stimulating current for non-planar electrodes (Fig. 10(c), (d)). At ERD = 40 μm, the RGC areas activated by the five differently shaped electrodes were compared, as shown in Fig. 10(e). Under stimulation with the same current (1.25 × TC), the concave electrodes had the smallest activated RGC area (0.013 mm^2^), followed by the disk electrode (0.018 mm^2^), the convex hemispherical electrode (0.022 mm^2^), and the convex conical electrode (0.023 mm^2^).

### The edge effect

**Fig. 11:**
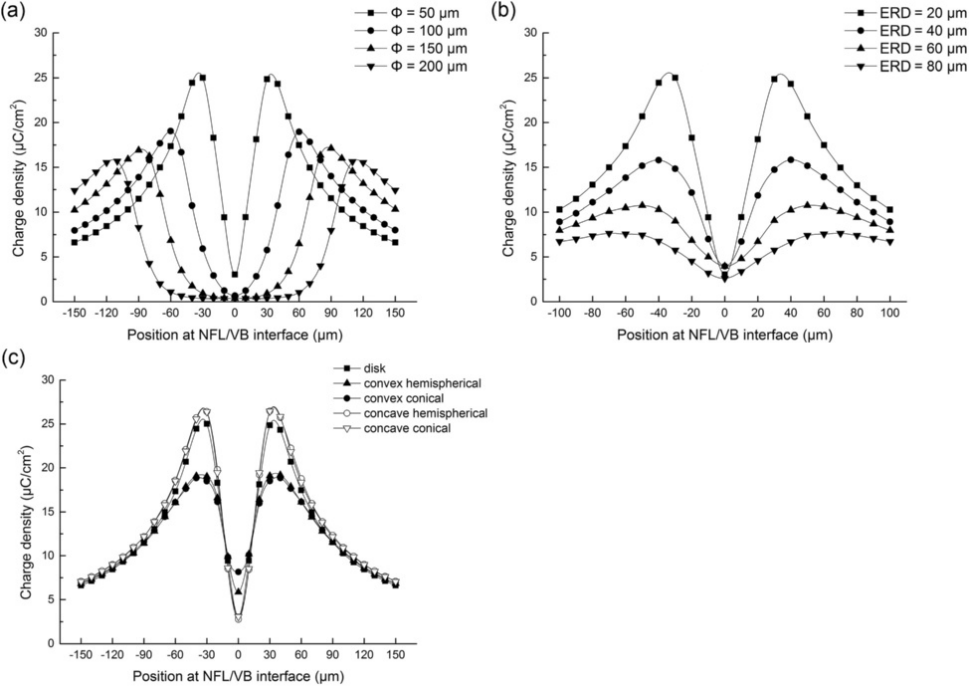
Charge density distribution at the NFL/VB interface under electrical stimulation of TC. **a** Correlation between disk electrode size and the distribution of charge density. The diameter of disk electrode varied from 50 μm to 200 μm, and all ERD was fixed at 20 μm. (TC: Φ = 50 μm: 2.15 μA; Φ = 100 μm: 2.61 μA; Φ = 150 μm: 3.37 μA; Φ = 200 μm: 4.05 μA) (**b**) Correlation between ERD and the distribution of charge density. The ERD varied from 20 μm to 80 μm, and the diameter of disk electrode was fixed at 50 μm. (TC: ERD = 20 μm: 2.15 μA; ERD = 40 μm: 3.66 μA; ERD = 60 μm: 5.07 μA; ERD = 80 μm: 6.18 μA) (**c**) Correlation between the electrode geometrical shape and the distribution of charge density. All electrodes were with the same 50 μm diameter disk projection to the retinal plane and the same ERD of 20 μm. (TC: disk: 2.15 μA; concave hemispherical: 4.27 μA; concave conical: 4.26 μA; convex hemispherical: 6.78 μA; convex conical: 6.74 μA)

To investigate the edge effect’s influence on electric stimulation safety, we calculated the charge density distribution on the inner retinal surface (i.e. NFL/VB interface) under electrical stimulation with the TC. Figure 11(a), (b) illustrate the charge density distributions when disk electrodes stimulated the retina with varying Φ and ERD. These results show that the edge effect was more significant for smaller disk electrodes and shorter ERD. Figure 11(c) shows the comparisons of charge density distribution among the disk and 3-D electrodes. The concave and disk electrodes had much sharper changes in charge density than the convex electrodes, and the ratio of charge density peaks between the concave conical and convex conical electrodes was 2.24.

## Discussion

The design of stimulating electrodes plays a crucial role in the performance of epiretinal prosthese. Model-based simulation on epiretinal electrical stimulation provide useful support for electrode design in the future. The objective of this study is to evaluate the effects of ERD, the shape and size of different 3-D electrodes on RGC excitation through the use of computational analysis. Thus, we built a retinal stimulation model in COMSOL and retinal ganglion cell excitation model in NEURON. For disk electrodes, the effects of ERD and electrode size were studied. Furthermore, the impacts of 3-D electrodes with different geometrical shapes were also investigated. Finally, the influence of edge effect on electric stimulation safety was discussed.

The spatial activated shapes and areas in RGCs are supposed to be relevant to the visual percepts or phosphenes perceived by the subjects implanted with visual prostheses in clinical trials [24]. In our results, the shapes of the activated RGC areas were nearly elliptic, which was consistent with clinical results [34, 35]. The offset of the centers of the activated RGC areas can be explained by the fact that the high-density sodium-channel band - the most voltage-sensitive area of the ganglion cell - is located on the axon about 40 μm away from the soma.

Stimulation localization and electrode safety are two important factors in evaluating the performance of different electrodes. Stimulation localization represents the ability of electrodes to excite localized RGCs without activating the others, and it was evaluated by the activated RGC area under stimulation with the same TC multiple (for example, 1.25 × TC) in this study. Electrode safety depends on the stimulating charge density. To avoid tissue damage by electric stimulation, several electrochemical safety limits have been proposed. Previously, the most commonly used safe charge density limit was 0.35 mC/cm^2^ [36], and more recently, a limit as low as 0.1 mC/cm^2^ for cathodic stimulation with platinum electrodes has also been recommended [37]. The charge densities used in our study were all below 0.1 mC/cm^2^.

### Effects of the ERD

TC and TCD increased linearly with the increase of ERD (Fig. 7(e)). These phenomena were also observed in the simulation work conducted by Kasi et al. [38] and the electrophysiological experiments on rabbits by Jensen et al. [39]. Thus, electrodes are safer at a smaller ERD because the required TCD to excite RGCs is smaller than that at a larger ERD. Compared with Kasi et al. [38], the TCs in our stimulation were smaller, which may be due to the involvement of the high-density sodium-channel band in our model.

When stimulating RGCs with the same TC multiple, the activated RGC area was more concentrated when the electrode was placed near the retina. Therefore, an electrode with a small ERD would have better stimulation localization.

### Effects of disk electrode size

Smaller electrodes had lower TCs (Fig. 8(e)), since the electric fields were more centrally distributed above small electrodes [40]. However, the trend in TCD was opposite to that of TC: smaller electrodes had larger TCDs due to their small surface areas (Fig. 8(e)). These phenomena were also reported in the in-vitro electrophysiological experiments on rabbits by Sekirnjak et al. [33].

Under stimulation with the same TC multiple, the activated RGC area was more concentrated for a smaller electrode, indicating a better stimulating localization. Consequently, to achieve higher localization and lower TC, the stimulating electrode should be as small as possible. However, considering the higher charge density for small electrodes, which may reduce the safety of electrical stimulation, a compromise must be made between stimulation localization and electrode safety.

### Effects of electrode geometrical shape

We first compared the influences of concave electrodes on TC and TCD with disk electrode. At the same ERD, the concave electrodes had higher TCs than the disk electrode (Fig. 10(a)). However, with a given smaller ERD, the concave hemispherical and disk electrodes had the lowest TCDs, and the TCD for the disk electrode was the most sensible to ERD variations. When the ERD was increased to 80 μm, both the concave hemispherical and concave conical electrodes had lower TCDs than the disk electrode (Fig. 10(b)). This was probably due to the disk electrode’s smaller surface area. The concave hemispherical electrode had a lower TCD than the concave conical electrode, since the surface area of the hemispherical electrode was larger than that of the conical one. In summary, a disk electrode would be more efficient than a concave electrode due to the lower TC, but a concave hemispherical electrode would have better electric safety than a disk electrode when the ERD is larger than 40 μm. Under electrical stimulation with the same TC multiple, the activated RGC areas were the smaller for the concave electrodes, which indicated better localization of electrical stimulation. This is in accordance with the results of Djilas et al., who found that a concave, well-like electrode had good stimulation localization [27].

The effects of the convex electrodes on TC and TCD were compared with the disk electrode as well. The TCs of the convex electrodes were higher than that of the disk electrode at the same ERD. The TCDs of the convex electrodes were also larger than that of the disk electrode at a smaller ERD. However, as ERD was increased to 80 μm, the TCD of the disk electrode became larger than that of the convex hemispherical electrode due to its greater sensitivity to ERD. These phenomena may still be closely related to the larger surface area of the 3-D convex electrodes. Under stimulation with the same TC multiple, the activated RGC areas of the convex electrodes were larger than that of the disk electrode, which indicated weaker stimulation localization.

In comparison with the convex electrode, the concave electrodes had lower TCs, and the TCDs were also lower for the concave electrodes at smaller ERD (Fig. 10(a), (b)). The relative comparisons of TCs among different electrodes in Fig. 10(a) could be explained by the distribution of electric field strength illustrated in Fig. 5. As shown in Fig. 5, with the same current input and ERD, the electric field of disk electrode was the strongest due to its smallest surface area and the shortest average ERD. With the same ERD, the relative position of the electrode to the retinal tissue varied with the electrode shape, and the average ERD for disk electrode was the shortest. The strongest electric field of disk electrode resulted in the lowest TC compared to other electrodes. The comparisons of TCs between concave and convex electrodes could be accounted for in a similar way. Moreover, Fig. 10(e) illustrates that under the same TC multiple, concave electrodes showed the most focused activation region of RGCs. Considering the most localized stimulation as well as acceptable TC and TCD, it was concluded that a concave electrode, especially a hemispherical electrode, might have an advantage over a convex or disc electrodes.

### The edge effect and electric stimulation safety

The edge effect is the phenomenon of charge concentration at the edge of the electrode due to the mutual repulsion of same-polarity charges in the electrode, which leads to an unequal distribution of charge density and electric field strength. This phenomenon may induce neural tissue damage during electrical stimulation. Under stimulation with the TC, the maximum charge densities of all the electrode were lower than 30 μC/cm^2^, which was below the safe charge density limit of 65 μC/cm^2^ for biological tissue [41]. However, some electrodes had better electric safety than others. As shown in Fig. 11(a), (b), using a larger electrode and increasing the ERD would decrease both charge density and the edge effect, thereby reducing the possibility of tissue damage. In addition, as seen in Fig. 11(c), the convex electrodes had smaller peak charge densities compared to the concave and disk electrodes, which suggests that the convex electrodes had better electric safety in terms of the edge effect.

## Conclusions

Good epiretinal prosthesis performance is mainly dependent on effective electrical stimulation, which must be achieved through electrode optimization. In this study, 3-D computational models were established to investigate the effects of the disk and non-planar electrodes on epiretinal electrical stimulation. Non-planar electrodes had larger TCs than the disk electrode. Compared to the disk electrode, concave electrodes had superior stimulation localization and electrode safety while convex electrodes performed relatively poorly. Among the five types of electrodes, the concave hemispherical electrode may be the ideal candidate, considering its good stimulation localization and electrode safety. These conclusions may be beneficial for the optimization of the future electrode design.

## Abbreviations

RGC: Retinal ganglion cell
NFL: Nerve fiber layer
GCL: Ganglion cell layer
IPL: Inner plexiform layer
INL: Inner nuclear layer
OPL: Outer plexiform layer
ONL: Outer nuclear layer
OS: Outer segment
RPE: Retinal pigment epithelium
VB: Vitreous body
Pt: Platinum
PI: Polyimide substrate
FEM: Finite element method
ERD: Electrode-retina distance
TC: Threshold current
TCD: Threshold current density
